# Mapping and validation of *Fusarium* wilt race 2 resistance QTL from *Citrullus amarus* line USVL246-FR2

**DOI:** 10.1007/s00122-024-04595-z

**Published:** 2024-03-31

**Authors:** Venkata Rao Ganaparthi, Patrick Wechter, Amnon Levi, Sandra E. Branham

**Affiliations:** 1 Coastal Research and Education Center, Clemson University, Charleston, SC USA; 2 USDA, US Vegetable Laboratory, ARS, 2700 Savannah Highway, Charleston, SC 29414 USA

## Abstract

**Key message:**

Fon race 2 resistant QTLs were identified on chromosomes 8 and 9. Families homozygous for resistance alleles at a haplotype of three KASP markers had 42% lower disease severity than those with susceptible alleles in an independent, interspecific validation population confirming their utility for introgression of Fusarium wilt resistance.

**Abstract:**

*Fusarium oxysporum* f. sp. *niveum* (*Fon*) race 2 causes *Fusarium* wilt in watermelon and threatens watermelon production worldwide. Chemical management options are not effective, and no resistant edible watermelon cultivars have been released. Implementation of marker-assisted selection to develop resistant cultivars requires identifying sources of resistance and the underlying quantitative trait loci (QTL), developing molecular markers associated with the QTL, and validating marker-phenotype associations with an independent population. An intraspecific *Citrullus amarus* recombinant inbred line population from a cross of resistant USVL246-FR2 and susceptible USVL114 was used for mapping *Fon* race 2 resistance QTL. KASP markers were developed (*N* = 51) for the major QTL on chromosome 9 and minor QTL on chromosomes 1, 6, and 8. An interspecific F_2:3_ population was developed from resistance donor USVL246-FR2 (*C. amarus*) and a susceptible cultivar ‘Sugar Baby’ (*Citrullus lanatus*) to validate the utility of the markers for introgression of resistance from the wild crop relative into cultivated watermelon. Only 16 KASP markers segregated in the interspecific *C. amarus/lanatus* validation population. Four markers showed significant differences in the separation of genotypes based on family-mean disease severity, but together explained only 16% of the phenotypic variance. Genotypes that inherited homozygous resistant parental alleles at three KASP markers had 42% lower family-mean disease severity than homozygous susceptible genotypes. Thus, haplotype analysis was more effective at predicting the mean disease severity of families than single markers. The haplotype identified in this study will be valuable for developing *Fon* race 2 resistant watermelon cultivars.

**Supplementary Information:**

The online version contains supplementary material available at 10.1007/s00122-024-04595-z.

## Introduction

Watermelon is an important specialty crop in the USA but domestic production has decreased over the last few decades. Watermelons were produced on 102,400 acres in 2021, with a gradual annual decrease from the maximum domestic production of 220,000 acres in 1992 (NASS^ 2022^). Correspondingly, imports have steadily increased to meet steady domestic demand (USDA^ 2020^). One of the major reasons for decreased acreage and production is yield losses from soil-borne diseases, especially *Fusarium* wilt. *Fusarium* wilt is caused by four races of the fungal pathogen *Fusarium oxysporum* f. sp. *niveum* (*Fon*), i.e., race 0, race 1, race 2, and most recently, race 3 (Martyn 2014; Petkar et al. 2019). *Fon* race 2 (*Fon*R2) is the most common race in South Carolina soils (Keinath et al. 2020) and has spread worldwide over the last three decades (Bruton et al. 2008; Egel et al. 2007; Gonzalez‐torres et al. 1993). Commercial cultivars with resistance to *Fon* races 0 and 1 are widely available; however, there are no edible cultivars with resistance to races 2 or 3. Integrated disease management strategies incorporating the usage of these resistant cultivars have greatly controlled losses due to race 1 (Martyn 2014). Discontinuation of the highly effective fumigant methyl bromide due to a mandate by the Montreal Protocol and Clean Air Act (1998) eliminated the most effective chemical management for *Fusarium* wilt. Other chemicals such as prothioconazole and thiophanate-methyl were shown to reduce the severity of watermelon *Fusarium* wilt caused by *FonR2*; however, they do not prevent economic losses (Everts et al. 2014). Management practices such as cover cropping with *Vici*a *villosa*, *Trifolium incarnatum*, *Secale cereale* and *Brassica juncea* reduced *Fusarium* wilt on triploid watermelon by 2 to 21% (Himmelstein et al. 2014). Grafting susceptible watermelon scions onto resistant rootstocks reduces disease severity and is currently the most effective management strategy against *FonR2* (Keinath and Hassell 2014), but increases the cost of cultivation. Developing resistant edible *Citrullus lanatus* cultivars coupled with an effective integrated disease management plan will increase profits for growers through decreased yield losses and lower cost of chemical controls.

Apart from two Indian *C. lanatus* accessions with higher seedling survivability, no resistance has been reported in cultivated watermelons (Pal et al. 2023). Screening of the USDA *Citrullus amarus* (*C. amarus*) plant introduction (PI) collection identified a few accessions with high levels of resistance to *FonR2* (Wechter et al. 2012). *C. amarus* and *C. lanatus* are easily crossable allowing exchange of genes between two species (Ren et al. 2015). Inbred lines, USVL246-FR2 and USVL252-FR2, were developed from the two most resistant *C. amarus* PIs (Wechter et al. 2016). QTL mapping with bi-parental early generation (F_2:3_) *C. amarus* population identified one major (on chromosome 9) and four minor QTLs associated with *FonR2* resistance (Branham et al. 2017). The need to phenotype numerous individuals for each F_3_ family to estimate the true genetic effect of the respective F_2_ for a quantitative trait makes performing such large bioassays resource prohibitive. This limitation can lead to under- or over-estimating discovered QTL effects compared with studies using recombinant inbred line (RIL) populations (Bernardo 2020). Theoretically, 50% of the markers in F_2_ populations are heterozygous. Double crossover and single crossover events between neighboring markers inherited from different homozygous parents (resistant and susceptible) within segregating individuals cannot be distinguished from non-crossover events leading to sparse genetic maps or inaccurate genetic distances. Conversely, all the genotyped parental polymorphisms can be utilized to estimate genetic distances in a RIL population (Paran et al. 1995). In Branham et al. (2019a, b), QTL mapping with a RIL population provided improved resolution and more reliable QTL effects for *Fon* race 1 resistance compared to the F_2:3_ population from which the RIL was derived. Further, lower resolution and often over-estimation of QTL effects with F_2:3_ populations decrease the effectiveness of marker development for marker-assisted breeding (MAS) (Austin and Lee 1996). We have generated an intraspecific *C. amarus* RIL population through single-seed descent of the F_2:3_ population previously used for mapping *FonR2* resistance (Branham et al. 2017). The RIL population was used in this study to identify/verify QTL to be targeted for MAS and to develop kompetitive allele-specific primers (KASP) tightly linked to *FonR2* resistance.

Given the availability of molecular markers strongly correlated with resistance, MAS can be implemented effectively in quantitative disease resistance breeding (Yeo et al. 2022; Ganaparthi et al. 2023a). However, use of MAS for trait improvement has yielded ambiguous results in different breeding programs (Damien et al. 2019). Accurate estimation of correlation between developed molecular markers and phenotype in elite genetic backgrounds for planned introgressions plays a critical role in the success of MAS or marker-assisted backcross selection (MABS) projects (Cobb et al. 2019). Thus, before using molecular markers in MAS or MABS for quantitative trait improvement, validation of the developed molecular markers in elite genetic backgrounds is warranted. The objectives of this study were to (1) validate and improve the resolution of *FonR2* resistance QTL from USVL246-FR2 in an intraspecific RIL population, (2) develop KASP markers tightly linked to the targeted QTL, (3) validate the developed KASP markers with an interspecific population between USVL246-FR2 (*C. amarus)* and the watermelon cultivar ‘Sugar Baby’ (*C. lanatus*), and (4) identify markers and/or haplotype(s) that can be used for MAS or MABS.

## Materials and methods

### Plant materials and growth conditions

An F_8:9_ RIL population (*N* = 200 lines) segregating for *FonR2* resistance, developed from resistant parent USVL246-FR2 and susceptible parent USVL114 (Branham et al. 2019a, b), was used for QTL mapping. An interspecific F_2:3_ population was developed from a cross of USVL246-FR2 with the *FonR2* susceptible cultivar ‘Sugar Baby’ (*C. lanatus*) for KASP marker validation. Eight F_1_ plants from the cross were self-pollinated by hand, and 161 randomly selected F_2_ seeds were germinated in 50-cell propagation trays (Hummert International, Earth City, MO). At the four-leaf stage, seedlings were transplanted into 6-L pots. Plants were maintained in the greenhouse at 25–35 °C. The second true leaf from each F_2_ plant was collected for DNA extraction using the CTAB method (Shu et al. 2018). Each F_2_ plant was selfed by hand-pollination to produce seeds for their respective F_3_ families. F_3_ families were phenotyped for disease response to artificial inoculation with *FonR2* and family means were used as phenotypes for their respective F_2_ individuals.

### Disease inoculations and evaluations

*FonR2* cultures were prepared using the optimized protocol described by Wechter et al. (2012). *FonR2* isolate B05-30cvd, obtained through single spore isolation, was grown on one-fourth Difco potato dextrose agar (Becton, Dickson & Co. Sparks, MD) for a week at 25 °C under 12-h diurnal light/dark cycle with fluorescent lighting. Five 1-cm disks were cut from the growing margin, added to 250 mL of potato dextrose broth, and placed on a rotary shaker at 200 rpm for two weeks at 25 °C temperature with 16/8-h light/dark cycle with fluorescent lighting. On the 15th day, the spore suspension was filtered through two layers of cheesecloth and a layer of Mira cloth (EMD chemicals, San Diego, CA) to remove hyphae. Spores were quantified by microscopy using a hemacytometer, and the final concentration was adjusted to 10^6^ cfu/mL with sterile distilled water. Five liters of the diluted spore suspension was added to 22 L of tri-mix (perlite (THERM-O-ROCK EAST, INC.): vermiculite (Palmetto vermiculite, SC): metromix 360 potting soil (Sun Gro Horticulture Inc) in 1:1:1 ratio). Spores were homogeneously distributed in the tri-mix using an electric concrete mixer. Ten seeds each of the 200 RILs, along with their parents, F_1_ and race differentials (Burton et al. 2008) were evaluated in two tests with two replications (five seeds per each rep) per test. Seeds were seeded into inoculated tri-mix in 50-cell propagation trays and were grown in a growth chamber with LED lights (4 red: 1 blue) at 25 °C. All the plants grown in growth chambers were subjected to a 16:8 light-to-dark photoperiod. Similarly, 10 seeds per each of the 161 F_2:3_ families were evaluated in two tests into inoculated soil and were grown in a growth chamber with LED lights (4 red:1blue) maintained at 25 °C. Plants in each test were rated on a 1–5 scale on the 28th day after planting in inoculated soil as described by Ganaparthi et al. (2023b). Completely healthy plants were rated as 1 and plants with turgid stems but with chlorotic or necrotic spots either on cotyledons or true leaves were given a rating of 2. Plants with chlorotic or necrotic spots on both cotyledons and true leaves with turgid stem were rated as 3. Completely wilted plants were rated as 4 and completely necrotic plants were rated as 5.

### Statistical analysis

Best linear unbiased estimates (BLUEs) for each test separately and the two tests combined were obtained for each RIL using the lmer function in the R package lme4 (R core team 2022) and were utilized in the mapping experiment.

The model utilized for obtaining BLUEs was:

$$Y= {g}_{i}+{r}_{j}+{t}_{k}+{g}_{i}:{t}_{k}+{e}_{ij}$$

where *Y* represents the BLUEs of each accession, ${g}_{i}$ is the fixed effect of the ith genotype and ${r}_{j}$ is the random effect of the jth rep, ${t}_{k}$ is the random effect of the kth test, ${g}_{i}: {t}_{k}$ is the interaction between ith genotype and kth test, and ${e}_{ij}$ is the random error variance. *FonR2* resistance broad-sense heritability among the mapping population was estimated using the formula (Piepho and Möhring 2007):

$$H^{2} =\frac{{\sigma }_{g}^{2}}{{\sigma }_{p}^{2}}$$

$${\sigma }_{p}^{2}= {\sigma }_{g}^{2}+\frac{{\sigma }_{gt}^{2}}{m}+\frac{{\sigma }^{2}}{rm}$$

where ${\sigma }_{g}^{2}$ is the variance due to genotype, ${\sigma }_{p}^{2}$ is the phenotypic variance, ${\sigma }_{gt}^{2}$ is the variance of the genotype-by-test interaction, $m$ is the number of tests and $r$ is the total number of replicates. Homogeneity of test and replication variances were tested with Bartlett’s test. Disease severity means for each F_2:3_ family in the validation population were utilized in identifying significant molecular markers or haplotypes correlated with *FonR2* resistance.

### Genetic map construction and QTL mapping

The SNPs (*N* = 2,143) and genetic map of the RIL population were obtained from Branham et al. (2019a, b) and were utilized for the initial mapping of *FonR2* QTL(s) with the R package qtl (Broman et al. 2003). KASP markers were developed from the identified QTL (described below) and used to genotype the RIL population. KASP SNPs with identical segregation patterns were binned and then combined with the GBS SNPs to make a new genetic map (in Rqtl) for QTL mapping to improve resolution.

Linkage groups were formed with the formlinkagegroup function using a maximum recombination frequency of 0.35 and with a minimum LOD score of 7. Markers that did not fit into any of the 11 linkage groups were removed from the genetic map. Using the Kosambi mapping function, genetic distance between the marker pairs were calculated based on the recombination fraction between them (Kosambi 1943). Multiple QTL mapping (MQM) with Haley–Knott regression (Haley and Knott 1992) was used to identify potential genomic regions imparting *FonR2* resistance in the RIL population. The optimal model with the highest penalized LOD score was determined with the stepwise qtl function (Broman and Sen 2009). The LOD value significance threshold was determined with 1,000 permutations using scantwo function with penalties at $\alpha =0.05$. LOD profile figures were generated with the scanone function, and the addqtl function was used to add single additional QTL to visualize forward model selection. The Lodint function with argument ‘expandtomarkers = T’ identified the markers flanking each significant QTL for the 1.5 LOD interval. USVL246-FR2 genome annotations (Wu et al. 2023) were obtained from the CuGenDBv2 database (Yu et al. 2022) and used to identify the candidate genes within the 1.5 LOD interval of significant QTL.

### KASP marker development and DNA amplification conditions

KASP markers were developed for all the significant *FonR2* resistance QTL (Supplementary Table 1). The physical position of SNPs within the 1.5 LOD intervals of significant QTL were obtained from USVL246-FR2 v1 reference gene annotations (Wu et al. 2023). To design KASP suitable for interspecific introgression into *C. lanatus*, flanking genomic sequence (60 bp up- and downstream) were obtained and queried using BLAST against the reference genome of 97,103 v 2.5 (Guo et al. 2013), a *C. lanatus* elite line susceptible to *FonR2*. BLAST searches where all flanking nucleotides were monomorphic and the targeted SNP was polymorphic between resistant and susceptible individuals were selected so that KASP could be \used for interspecific introgression. Alternate alleles of the selected SNPs, along with respective flanking sequences, were sent to either LGC Genomics (Hoddesdon herts, UK) or Integrated DNA Technologies (Beverly, MA, USA) for designing primers. A total of 51 KASP markers across the major and minor resistance QTL intervals were developed from SNPs identified between the parents and sequence similarity to the *C. lanatus* reference genome. PCR reactions for amplification included 2.5 μL of Master mix (LGC Genomics, Alexandria, MN), 0.07 μL of primer mix (two fluorophore labeled allele-specific forward and a reverse primer), and 15–20 ng of sample DNA brought up to a 5 μL reaction with DI water. A touchdown PCR protocol was programmed on a standard thermal cycler beginning with a 15 min activation step at 94 °C followed by 11 alternate cycles of denaturation and annealing, 20 s cycles at 94 °C for denaturation, and initial annealing at 61 °C reduced by 0.6 °C each cycle for 60 s. These touchdown cycles were followed by 26 additional alternate cycles of denaturation and anneling at 94 °C for 20 s and 55 °C for 60 s, respectively. A Stratagene Mx300P (Agilent technologies, Santa Clara, CA) quantitative PCR system was used to quantify fluorescence for allele discrimination. Samples were clustered with MxPro v4.10 software (Agilent technologies, Santa Clara, CA) based on normalized HEX and FAM readings. Parents and the RIL population were genotyped with all the KASP markers, and only markers that showed polymorphism between the parents were utilized in genotyping the USVL246-FR2 x ‘Sugar Baby’ population. KASP markers significantly associated with resistance were identified with analysis of variance (ANOVA) of each marker on family-mean disease severity. R^2^ explained by all significant markers on regression with family mean was reported as the phenotypic variance explained. All possible haplotypes with significantly associated markers were identified. The significance of all families homozygous for the resistant or susceptible parent haplotype group was assessed with a Welch two-sample t test.

## Results

### Genetic map

A total of 2,185 SNPs were obtained for the intraspecific RIL population after combining variants from GBS and KASP. After dropping markers with identical segregation patterns, 2,177 SNPs were used for genetic map construction. Three markers did not fit into any linkage groups and were removed from the analysis as they are likely to be genotyping errors. Thus, 2,174 markers were left for further analysis. The total genetic map length was 1,192.9 cM with an average spacing of 0.6 cM between markers (Supplementary Table 1). Linkage groups were named according to the chromosomes of the USVL246-FR2 *C. amarus* reference genome (Wu et al. 2023). The maximum spacing between markers was 21.3 cM on chromosome 8. Chromosome 10 spanned 137 cM and was the largest chromosome, while chromosome 4 was the shortest with a length of 84 cM.

### *FonR2* resistance in the intraspecific RIL population and interspecific F_2:3_ population

**Fig. 1:**
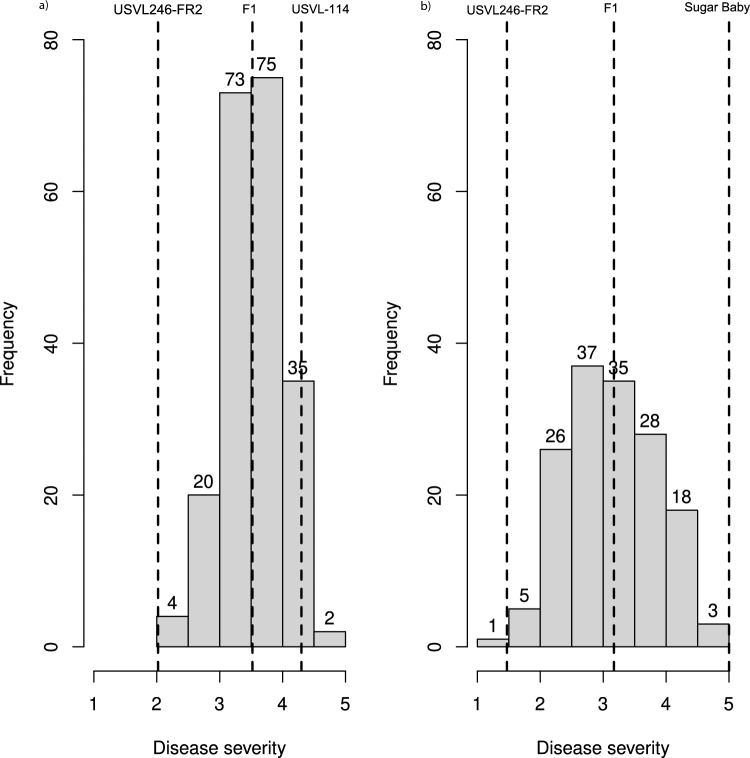
Histograms showing the disease severity distributions for: **a** USVL246-FR2 X USVL-114 RIL population across two tests, **b** USVL246-FR2 X Sugar Baby F_3_ families inoculated with *FonR2*. Parental and F_1_ means are marked by vertical dashed lines. Numbers on each bar indicates number of RILs or F_3_ families in specific range of disease severity

Disease severity variances for test 1 and test 2 of the intraspecific population were non-homogenous (*P*-value is 4.38e-09). Pearson correlation between BLUEs of test 1 and test 2 was 0.41. Replications within test 1 were non-homogenous (*P*-value is 0.0028), while replications within test 2 were homogenous. The coefficient of variance (CV) for test 1, test 2 and across the two tests was 0.17, 0.16 and 0.13, respectively. The mean disease severity of the RIL population across the two tests was normally distributed (Fig. 1). The mean disease severity of resistant and susceptible parents of the mapping population was 2.0 and 4.4, respectively. The mean disease severity of the RILs ranged from 2.1 to 4.5 across two tests, and the mean disease severity of the F_1_ was 3.5. Overall mean disease severity of the RIL population was 3.6. Broad sense heritability of disease severity in the RIL population was 0.57. Analysis of variance determined genotype and test as highly significant (Table 1).

**Table 1:** Analysis of variance of *FonR2* disease severity in 200 RILs evaluated in the growth chamber

Factor	Sum sq.^a^	F value	*P*-value
Genotype	178.28	2.391	7.16e-14***
Test	85.41	231.362	< 2e-16***
Rep(test)	15.61	21.141	1.87e-09***
Genotype: test	84.90	1.150	0.123
Residuals	244.97	NA^d^	NA

^a^Sum of squares

^***^Significant at 0.001 level of significance

The mean disease severity of the interspecific F_2:3_ population was 3.2, which was lower than the RIL population (3.6). Pearson correlation between means of test 1 and test 2 was 0.54. The lowest family mean of disease severity in the F_2:3_ population was 1.3, and the highest was 4.9 indicating transgressive segregation towards the resistant parent (Rieseberg et al. 1999) (Table 1). Broad sense heritability of disease severity in the F_2:3_ interspecific population was 0.32.

### QTL mapping

**Fig. 2:**
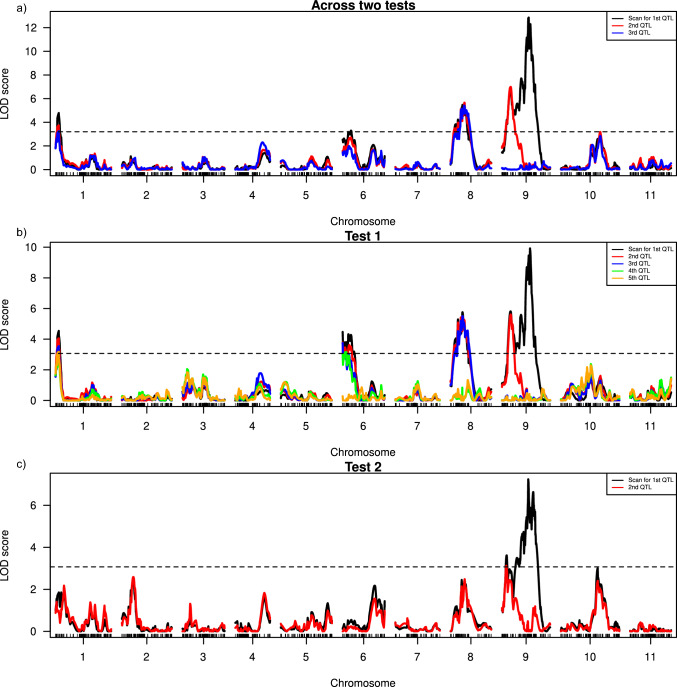
Logarithm of odds (LOD) scores for QTL associated with best linear unbiased estimates (BLUEs) obtained from disease severity **a** Across two tests **b** Test 1 **c** Test 2 after inoculation with *FonR2*. The horizontal dashed line indicates the genome-wide significance threshold

**Fig. 3:**
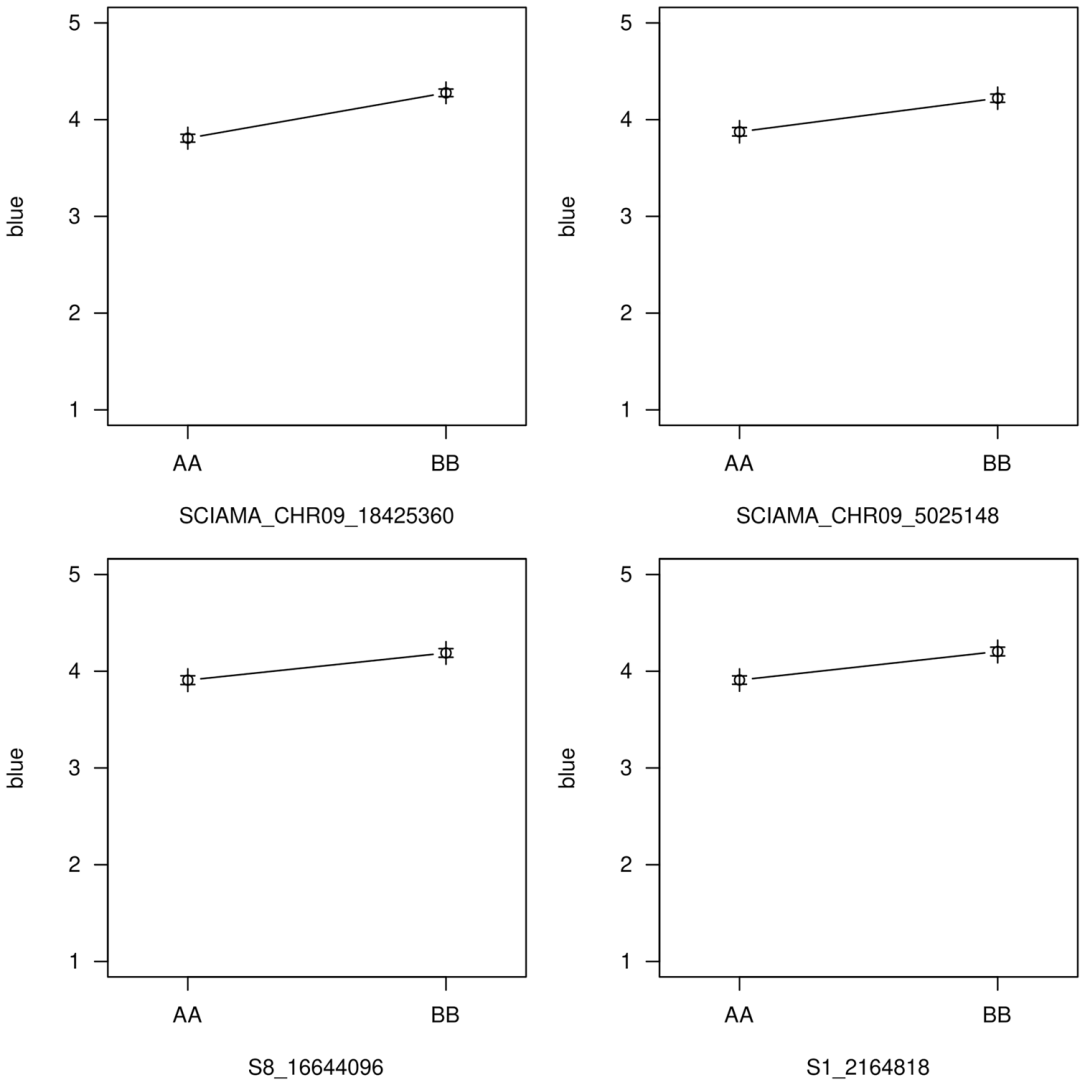
Effect plots showing the mean and standard errors for best linear unbiased estimate of *Fusarium oxysporum* f. sp. *niveum* race 2 disease severity for genotypic class: AA homozygous resistant parent alleles, BB homozygous susceptible parent alleles. Panels depict the genotype of the SNP with highest LOD score for each quantitative trait loci identified across two tests: **a** SNP at QTL *qFon2-9.2*; **b** SNP at QTL *qFon2-9.1*; **c** SNP at QTL *qFon2-8*; **d** SNP at *qFon2-1*

The optimal multiple QTL mapping (MQM) model with the highest penalized LOD score was determined for each test and across tests. MQM of BLUEs across two tests identified three QTLs as significantly associated with *FonR2* resistance in the intraspecific RIL population. Of the three QTLs, two were identified on chromosome 9 and one on chromosome 8 (Fig. 2a). The QTL with the highest LOD score was identified at 62.5 cM on chromosome 9, explaining 20.7% of the phenotypic variance with an additive effect of 0.22 (Table 2). Two minor QTLs, on chromosomes 9 and 8, explained 9.4 and 7.5% of the phenotypic variance, respectively. No epistatic interaction between QTL was identified. MQM of test 1 BLUEs identified 5 QTL associated with *FonR2*. The major QTL on chromosome 9 had a peak LOD score (8.85) at 65.6 cM and explained 12.0% of the phenotypic variance. Four minor QTLs were identified on chromosomes 9, 8, 6 and 1. Together, the minor QTL explained 21.6% of the total phenotypic variance. However, MQM with BLUEs obtained from test 2 identified only two significant QTL, both on chromosome 9 and collocated with the two QTL identified on chromosome 9 from test 1. KASP markers were developed for the major QTL on chromosome 9 and the minor QTL on chromosomes 8, 6 and 1 (Table 2) to pyramid resistance QTL for *FonR2* resistance after their validation (Fig. 3).

**Table 2:** Quantitative trait loci (QTL) associated with disease severity within each test and across tests after inoculation with *FonR2*

Test	QTL	Chromosome	Peak (cM)	Range (Mb)^a^	Peak LOD score	% V_p_^b^	Additive^c^
1	qFon2-1	1	8.3	0.11 – 2.55	3.18	4.04	0.13
1	qFon2-6	6	6.5	0.63 – 4.94	3.20	4.05	0.13
1	qFon2-8	8	30	7.04 – 17.71	5.42	7.06	0.17
1	qFon2-9.1	9	21.0	3.83 – 7.15	5.00	6.48	0.17
1	qFon2-9.2	9	65.6	13.8 – 26.12	8.86	12.00	0.22
2	qFon2-9.1	9	9.5	1.68 – 5.6	3.12	5.92	0.12
2	qFon2-9.2	9	61.5	15.85 – 30.47	6.83	13.53	0.18
Across two tests	qFon2-8	8	35	14.83 – 18.81	5.47	7.50	0.13
Across two tests	qFon2-9.1	9	19.0	3.83 – 5.6	6.76	9.40	0.15
Across two tests	qFon2-9.2	9	62.0	15.85 – 25.9	13.68	20.66	0.22

^a^Physical region of the genome corresponding to 1.5-LOD QTL interval

^b^Percent of phenotypic variation explained by the QTL

^c^Additive effect of the QTL

### KASP marker validation

**Fig. 4:**
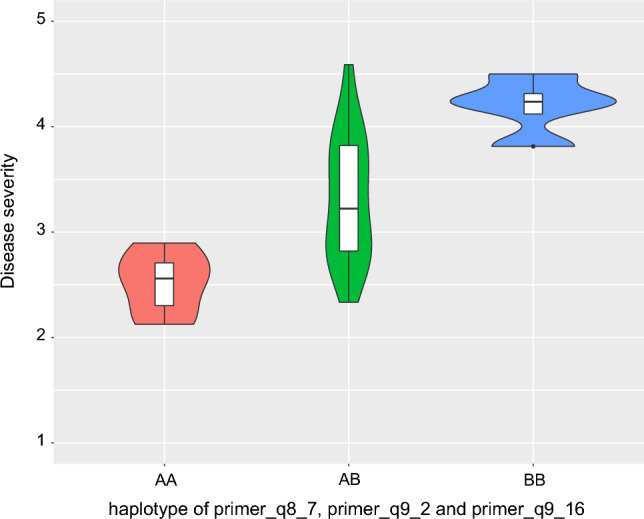
Violin plot showing mean disease severity distribution of F_2:3_ families that inherited resistant (**AA**), resistant and susceptible (**AB**) and susceptible (**BB**) parent alleles at primer_q8_7, primer_q9_2 and primer_q9_16 with their respective group median and confidence intervals. X-axis represents parent alleles inherited at haplotype loci

Four KASP markers on chromosome 9, eight on chromosome 1, six on chromosome 6 and eight on chromosome 8 were polymorphic between the parents, these markers were used for genotyping the interspecific F_2_ population. Nevertheless, only three KASP markers for each QTL on chromosome 9 and chromosome 1 and five markers each for QTL on chromosome 6 and chromosome 8 showed useful polymorphism among interspecific population. ANOVA of each KASP marker on family-mean disease severity identified four markers (primer_q1_1, primer_q8_7, primer_q9_2 and primer_q9_16) as significantly associated with *FonR2* disease severity. However, all significant markers combined explained only 16% of the phenotypic variance. To identify more meaningful association between markers and family-mean disease severity, all possible haplotype combinations with significant markers and their associated phenotypes were tested (Supplementary Table 1). While some haplotypes exhibited significant differences with Welch two-sample t test, they were not useful in discriminating resistant/susceptible families. One haplotype combination consisting of three KASP markers (q8-7, q9-2 and q9-16) was highly significant (*P*-value = 5.21e-05) and exhibited strong association (R^2^ = 0.42) between alleles and family-mean phenotype. Seven families that inherited resistant parental alleles at these three loci, had a mean disease severity of 2.51 and six families with susceptible parental alleles had a mean disease severity of 4.35 (Fig. 4). The mean disease severity of 13 heterozygous families for the haplotype was 3.23. Only one family inherited resistant parental alleles at all four significant loci and had a mean disease severity of 2.64.

## Discussion

Sources of resistance to *Fusarium* wilt caused by *FonR2* are limited (Wechter et al. 2012). The genetic architecture of resistance from a few of these sources was determined through QTL mapping with bi-parental mapping populations (Branham et al. 2017, 2020; Meru and McGregor 2016; Ren et al. 2015). QTL mapping studies using USVL252-FR2 as the resistance source identified QTL on chromosomes 1, 5, and 8 (Branham et al. 2020). Two QTLs, on chromosomes 9 and 10, were associated with *FonR2* resistance using PI296341-FR as the resistance source (Ren et al. 2015). In a study by Meru and McGregor (2016) using WGA147 as the resistance donor, a single resistance QTL was identified on chromosome 10. Quantitative trait nucleotides (QTNs) were also identified with a genome-wide association study of *Fonr2* resistance in the USDA *C. amarus* collection (Ganaparthi et al. 2023b). Ganaparthi et al. (2023b) identified five QTNs on chromosomes 1, 5, 9 and 10 associated with *FonR2* resistance.

Here, we identified a total of five QTLs associated with *FonR2* resistance, including two on chromosome 9 (*qFon2-9.1 and qFon2-9.2*), and one each on chromosomes 1 (*qFon2-1*), 6 (*qFon2-6*), and 8 (*qFon2-9.1*). QTLs *qFon2-*1 and *qFon2-9.1* collocate with those identified from other sources, while QTLs *qFon2-6, qFon2-8* and *qFon2-9.2* are novel. QTLs *qFon2-1* and *qFon2-6* were only significantly associated with resistance in test 1, therefore they were excluded from further consideration. None of the QTLs identified by (Branham et al. 2020) collocate with QTLs identified in the current study. The QTL identified on chromosome 9 by Ren et al. (2015) is physically close to the *qFon2-9.1* in the current study. The QTN identified on chromosome 9 through GWAS is 8.4 Mb upstream of *qFon2-9.2* (Ganaparthi et al. 2023b). Thus, two QTL (i.e., *qFon2-9.2* and *qFon2-*8) for *FonR2* resistance identified in this study are likely to be unique to USVL246-FR2. Along with *FonR2* resistance, USVL246-FR2 is also resistant to *Fusarium* wilt caused by race 1. The same RIL population was also used to map *Fon* race 1 resistance QTL (Branham et al. 2019a, b). The major resistance QTL *qFon2-9.2* overlaps for races 1 and 2 in this population (15.6 to 26.1 Mb and 15.85 to 25.9 Mb, respectively). Therefore, QTL *qFon2-9.2* either provides broad-spectrum *Fusarium* wilt resistance or the resistance QTLs for *Fon* races 1 and 2 are tightly linked.

The RIL population (USVL246-FR2 by USVL114) utilized in this study was derived from a previously published F_2:3_ population (Branham et al. 2017) through single-seed descent. *FonR2* resistance was associated with a single major QTL on chromosome 9 and four minor QTLs in both populations, however, differences between the results were found. In comparison with the mapping performed on the F_2:3_ population (Branham et al. 2017), the estimated effect of the major QTL was smaller, the QTL interval identified on chromosome 8 was narrowed by 8.8 Mb, and fewer QTLs were identified with the RIL population. The major QTL discovered with the F_2:3_ population, *qFon2-9,* explained 43% of the phenotypic variance. Two QTL (*qFon2-9.1* and *qFon2-9.2*) were identified on either side of *qFon2-9* in the RIL population and explained 30% of the phenotypic variance. QTL *qFon2-9* could be a ghost QTL: a spurious QTL that can arise when two QTLs are on the same chromosome (Martinez and Curnow 1992; Ronin et al. 1999; Stange et al. 2013). Lower recombination among the F_2:3_ population may have rendered the two QTLs closer in the genetic map causing them to appear as a single QTL with a large effect between the two QTL intervals.

Two hypotheses could explain the detection of more QTLs with the F_2:3_ than the RIL population. First, F_2:3_ populations include QTL with over-dominance effects, but those same QTL will not be identified using a RIL population. Another possibility is that lower recombination in the F_2:3_ population resulted in clustering of genomic regions with a small effect, causing r/qtl to detect a QTL at these clusters. Such clustered genomic regions can break apart due to higher recombination; thus, the minor effect QTL may not be detected with the RIL population (Austin and Lee 1996). In crops where hybrids are commercially grown and traits targeted for improvement exhibit both additive effects and over-dominance, mapping of QTL employing both F_2:3_ and RIL populations could be beneficial in devising breeding strategies for trait improvement. Finally, because of true replication due to homogeneity within each RIL, phenotype estimates in a RIL population are better than the estimates obtained for the segregating families in early generation populations. This improved precision in phenotypic estimation of genotypes along with a higher recombination rate allows for narrowed QTL intervals and lower phenotypic variances explained by significant QTL with RIL populations compared to F_2:3_ populations (Austin and Lee 1996). These two hypotheses also explain the higher heritability among RILs (0.57) compared with the F_2:3_ (0.32) population.

Marker validation studies with a *C. lanatus* population are required to demonstrate the effectiveness of resistance introgression into a cultivated watermelon background using MAS or MABS. Also, validation of markers developed for respective QTL in an independent population aid in construction of an effective model for genomic selection (Jannink et al. 2010; Poland and Rutkoski 2016; Rutkoski et al. 2014; Zhang et al. 2021). Although the *FonR2* KASP markers were checked for introgression utility by genomic comparison of the wild (*C. amarus*) and cultivated (*C. lanatus*) genomes, only a few (*N* = 16) of the markers developed were polymorphic in the segregating interspecific population. Four of the polymorphic markers were significantly associated with *FonR2* resistance in the interspecific validation population, including two markers within *qFon2-9.2*, one within *qFon2-8* and one near *qFon2-1*. The realized effects of these QTL (16%V_P_) are smaller than the estimated effect with the RIL population. Haplotype analysis identified a combination of three significant markers, from QTLs *qFon2-8, qFon2-9.1 and qFon2-9.2,* with improved association with disease severity as compared to single markers. Families with resistant parental alleles at these three loci exhibited 42% less disease severity than families with susceptible parental alleles, suggesting that the two QTLs are complementary. Few families inherited resistant parental alleles at these three loci and only a single genetic background (‘Sugar Baby’) was used to confirm the haplotype effect on lowering disease severity. Future work will focus on validating the utility of the haplotype in different genetic backgrounds (cultivars).

Functionally relevant candidate genes were found within each QTL. Ethylene receptors and transcription factors are essential in ethylene-mediated plant response to biotic stress (Müller and Munné-Bosch 2015). Over-expression of ethylene or endogenous application of ethephon induced resistance to *Fusarium oxysporum* f. sp. *cucumerinum (Foc*) in cucumber (Dong et al. 2020). Five ethylene transcription factors were identified within *qFon2-8*. Receptor-like kinases (RLKs) are well-known resistance genes (Goff and Ramonell 2007). RNA-seq data suggested that overexpression of lectin receptor-like kinases conferred resistance to *Fusarium* root rot in tomato (Yue et al. 2022). Transcriptome data showed evidence for RLK-induced *Fusarium* wilt resistance in cotton and Arabidopsis by recognition of *Fusarium* through its cell wall extract (Babilonia et al.^ 2021^). Genes predicted to encode RLKs were identified in the QTL intervals of *qFon2-9.*1 (*N* = 1 gene) and *qFon2-9.2* (*N* = 7 genes).

In summary, we used an intraspecific *C. amarus* RIL population to map four QTL associated with resistance to *FonR2*. KASP markers were developed across the QTL and validated in an interspecific F_2:3_ population of the resistant *C. amarus* line, USVL246-FR2, by the *C. lanatus* cultivar ‘Sugar Baby.’ Although four KASP markers across three chromosomes (1, 8 and 9) were significantly associated with resistance, a combination of three of these markers (on chromosomes 8 and 9) explained more of the phenotypic variance. Future work will focus on using this haplotype to introgress *FonR2* resistance into multiple elite backgrounds for development of improved watermelon cultivars.

### Supplementary Information

Below is the link to the electronic supplementary material.

Supplementary file1 (XLSX 99 KB)

Supplementary file 2 (Docx 11 KB)

## Data Availability

The datasets generated during and/or analysed during the current study are included in the supplementary tables.
