# Supplementary material for: Mapping and validation of Fusarium wilt race 2 resistance QTL from Citrullus amarus line USVL246-FR2
Source: Theor Appl Genet. 2024 Mar 31;137(4):91. doi: 10.1007/s00122-024-04595-z (PMC10982098; doi:10.1007/s00122-024-04595-z)
Supplement: Supplementary file 2 — (Docx 11 KB) [file 122_2024_4595_MOESM2_ESM.docx]

Supplementary materials

Supplementary table 1 Genetic map positions of SNP markers used in mapping of FonR2 resistance QTL in USVL246-FR2 x USVL-114 RIL mapping population.

Supplementary table 2 P-values showing significant marker-phenotype association and haplotype-phenotype association along with phenotypic variances.

Supplementary table 3 Sequences of KASP PCR markers utilized in genotyping the RIL population and F2:3 marker validation population.

Supplementary table 4 Genes from within 1.5 LOD interval of significant FonR2 resistance QTLs with BLUEs obtained from two tests along with gene ID.
